# Prediction of Class II improvement after rapid maxillary expansion in early mixed dentition

**DOI:** 10.1186/s40510-017-0163-3

**Published:** 2017-04-03

**Authors:** Alberto Caprioglio, Chiara Bergamini, Lorenzo Franchi, Nicolò Vercellini, Piero Antonio Zecca, Riccardo Nucera, Rosamaria Fastuca

**Affiliations:** 1 Division of Orthodontics, Department of Surgical and Morphological Sciences, School of Medicine, University of Insubria, Varese, Italy; 2 Division of Orthodontics, Department of Surgical and Morphological Sciences, Orthodontic Programme, School of Medicine, University of Insubria, Varese, Italy; 3 Division of Dentistry, Department of Surgery and Translational Medicine, University of Florence, Florence, Italy; 4 Department of Surgical and Morphological Sciences, School of Medicine, University of Insubria, Varese, Italy; 5 Division of Orthodontics, Department of Medical, Surgical and Health Sciences, University of Messina, Messina, Italy; 6 Department of Medical, Surgical and Health Sciences, University of Messina, Messina, Italy; 7 C/O Dental School, Via G. Piatti, 10, Velate, 21100 Varese, Italy

**Keywords:** Class II malocclusion, Maxillary expansion, Mixed dentition

## Abstract

**Background:**

The aim of this study is to identify cephalometric pretreatment parameters for prediction of Class II improvement induced by rapid maxillary expansion.

**Methods:**

Lateral cephalograms of 30 patients (mean age 8.3 ± 1.6 years old) showing Class II molar relationship and undergone to rapid maxillary expansion on the upper deciduous molars were traced before treatment, and molar relation changes were evaluated on dental casts before and after treatment. Overall treatment time lasted 10.2 ± 2 months. Good responders (18 subjects, 10 females and 8 males) showed improvement of at least 2.50 mm, and bad responders (12 subjects, 7 females and 5 males) showed no improvement, improvement less than 2.50 mm, or worsening of molar relationship after treatment. Student’s *t* test was used to assess significance of differences between groups, and discriminant analysis allowed identification of predictive pretreatment variables.

**Results:**

Articular angle, superior gonial angle, and mandibular dimensions (Co-Gn, S-Ar, Ar-Go, Go-Me) showed significant differences in the comparison between groups. Mandibular length Co-Gn and superior gonial angle were selected as significant predictive variable for discrimination.

**Conclusions:**

Patients with smaller mandibular length and more acute superior gonial angle are expected to have more chances to improve molar Class II after rapid maxillary expansion.

## Background

Distal relationship of the mandible to maxilla is usually described as Class II malocclusion, and it represents the most common disharmony in white race populations [1]. Either sagittal or vertical components were showed in Class II malocclusion patients; however, another relevant component is transverse dimension. Several authors [2, 3] evaluated transverse component of Class II malocclusion and found narrower maxillary arch in Class II division 1 malocclusion. Transverse maxillary deficiency, in fact, might not be evident in Class II patients due to occlusion of maxillary posterior teeth on narrower portions of the mandible [2, 3]. Indeed maxillary constriction might often be clinically observed by forcing lower jaw of Class II patients forward in dental Class I relationship. Tollaro et al. [4] found 3- to 5-mm narrower maxillary transverse dimension in Class II patients compared to ideal maxillary width relative to mandible without presenting posterior crossbite in centric occlusion. Franchi et al. [5] and Buschang et al. [6] showed that maxillary dental arch was narrower in Class II division 1 malocclusion compared to maxillary arch widths in normal occlusion in adult patients.

Based on previously reported findings, rapid maxillary expansion (RME) treatment was frequently suggested before Class II therapy [3, 4, 7–9]. Maxillary transverse deficiency might cause functional interferences, and removing maxillary constriction might lead to Class II spontaneous improvement. Even though improvement of dental Class II was showed after RME, disagreement was reported suggesting that RME might be detrimental for correction of Class II malocclusion, since the maxilla might be displaced downward and forward causing post-rotation of the mandible and then worsening Class II [10–12].

It was suggested that after RME treatment of the upper jaw, a “spontaneous” correction of Class II might take place due to forward posturing of the mandible to a more comfortable position [7, 8, 13]. McNamara [7] showed spontaneous improvement of dental Class II during retention phase of RME treatment in early mixed dentition patients. Disruption of occlusion and tendency to posture their jaw slightly forward improving sagittal occlusal relationships were reported [7]. In addition to variability in treatment response among different studies, similarly wide variability can be assessed within individual studies, i.e., a significant variability in response of individual patients to the same treatment protocol. The possibility to find any predictive variables might help the clinicians to distinguish favorable and unfavorable situations in order to provide when further correction of Class II malocclusion after RME would be needed.

The aim of this cephalometric investigation was therefore to identify possible pretreatment parameters for the prediction of individual Class II improvement induced by RME in early mixed dentition patients.

## Methods

The initial sample of the present retrospective study consisted of 122 Class II patients treated with RME selected from private practice (private practice Dr. A. Caprioglio, Pavia, Italy) and treated by the same trained operator (AC). Signed informed consent for releasing diagnostic records for scientific purposes was available from parents of patients. Among all patients only who satisfied inclusion and exclusion criteria were selected for the final group. Inclusion criteria were as follows: (i) Class II molar relationship described as end-to-end or full-cusp measured at the first permanent molars on both sides on dental casts; (ii) patients without discrepancy between centric relation (CR) and centric occlusion (CO); (iii) early mixed dentition (all first permanent molars erupted, as well as upper and lower permanent incisors and presence of all healthy deciduous molars) with stages 1 in cervical vertebral maturation (CVM); (iv) no other orthodontic or pediatric dentistry treatment; and (v) good general health (absence of craniofacial syndromes [14, 15] or other craniofacial anomalies [16]). Exclusion criteria were as follows: (i) unilateral/bilateral crossbite, asymmetrical Class II molar relationship, and/or open bite; (ii) loss of deciduous teeth during treatment; and (iii) use of other appliances before or during RME treatment.

From the initial sample of 122 patients, 30 patients (mean age 8.3 ± 1.6 years old; 13 males, 17 females) treated between January 2013 and December 2015 who satisfied inclusion and exclusion criteria were selected.

**Fig. 1:**
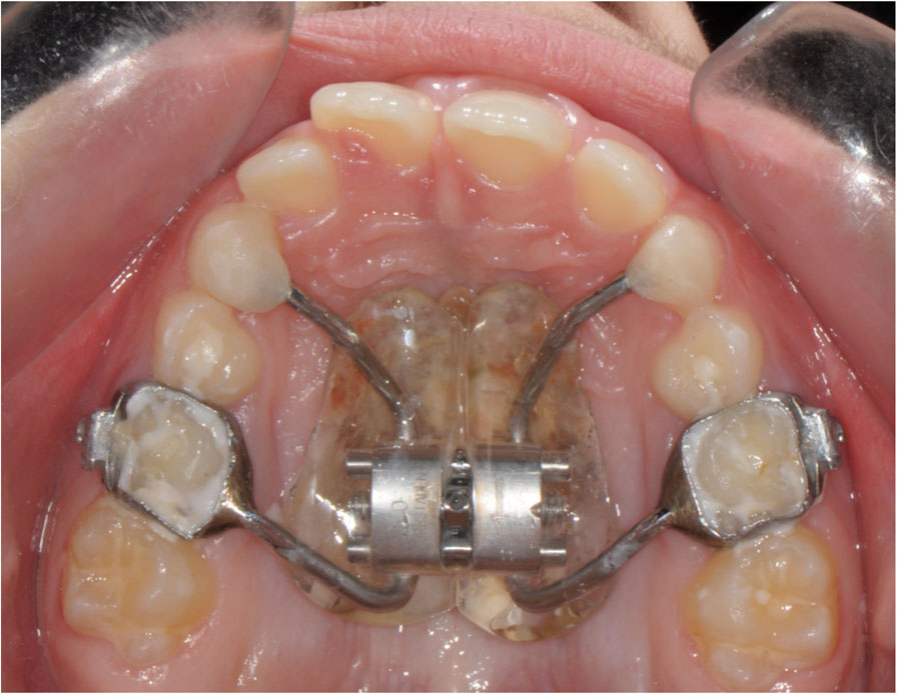
Haas-type expander on deciduous second molars

**Fig. 2:**
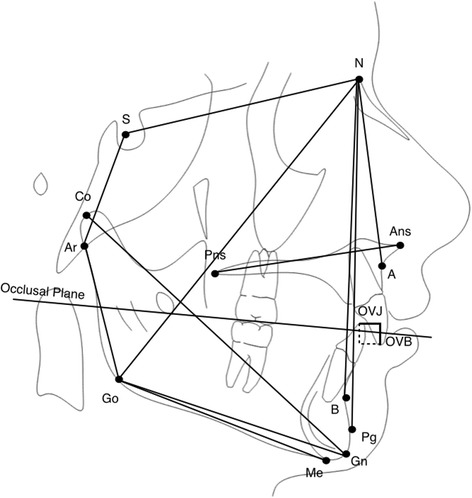
Cephalometric analysis. Description of the measurements in Table 1

Maxillary expander used for all subjects was Haas-type expander with a 10-mm screw (A167-1439, Forestadent, Pforzheim, Germany) banded to the upper second deciduous molars (Fig. 1). Maxillary expanders were banded using glass ionomer cement (Multi-Cure Glass Ionomer Cement, 3M-Unitek, Monrovia, CA) in accordance with the manufacturer’s instructions. The screw of the palatal expander was initially turned twice (0.45 mm initial transversal activation). Afterwards, parents of the patients were instructed to turn the screw once per each following day (0.225 mm activation per day). Maxillary expansion was performed until dental overcorrection, defined as when lingual cusps of the upper first molars occluded onto lingual side of buccal cuspids of the lower first molars, was achieved. The screw was then locked with light-cure flow composite (Premise Flowable; Kerr Corporation, Orange, CA), and expander was kept on the teeth as passive retainer. Overall treatment time lasted 10.2 ± 2 months. Study dental casts were available at the start (T1) and at the end (T2) of treatment. The information about the amount of expander activation was obtained from the patients’ diary. The mean activation of the screw was 6.25 ± 1.50 mm for the good responder (GR) group and 7.14 ± 1.27 mm for the bad responder (BR) group. At T2, discrepancy between CR and CO was checked again and the occlusion was registered with wax bite. Lateral cephalograms performed with the same X-ray machine in natural head position (NHP) [17–19] and by a single trained technician (AC) were available at the start of RME treatment for all patients (T1), were standardized as to magnification factor (6% enlargement), and were hand-traced by one single trained operator (CB) at the start of RME treatment (T1) (Fig. 2 and Table 1). Study dental casts and lateral cephalograms were all taken at the same time for all the patients.

**Table 1:** Cephalometric analysis

Sagittal measurements	
SNA (°)	
SNB (°)	
ANB (°)	
ANPg (°)	
Wits (mm)	
OVJ (mm)	
Vertical measurements	
AnsPns^GoGn (°)	
SN^GoGn (°)	
OVB (mm)	
Ar^Go^N (°)	
N^Go^Me (°)	
S^Ar^Go (°)	
N^S^Ar (°)	
Sum. Jaraback (°)	
Mandibular dimensions	
Co-Gn (mm)	
S-Ar (mm)	
Ar-Go (mm)	
Go-Me (mm)	

Eighteen cephalometric measurements (7 linear and 11 angular) were performed

### Definition of Class II improvement after RME treatment

Individual responsiveness of Class II malocclusion to RME treatment was defined on the basis of the T2-T1 improvement of Class II molar relationship described as end-to-end or full-cusp measured at first permanent molars on dental casts. The distance between mesio-buccal cusp of the first upper permanent molar and mesio-buccal cusp of the first lower permanent molar was measured with a digital caliper by a trained operator (CB). An improvement at least of 2.50 mm was considered, i.e., when a full cusp Class II molar relationship at T1 turned into an end-to-end at T2 or when an end-to-end at T1 turned into a Class I molar relationship at T2.

**Fig. 3:**
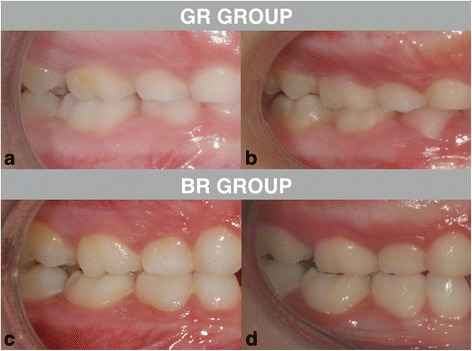
Examples of Class II molar relationship at T1 and T2 for good and bad responder. **a** Patient GR (*good responder*) at T1. **b** Patient GR at T2. **c** Patient BR (*bad responder*) at T1. **d** Patient BR at T2

On the basis of this reference, “GRs” were defined as those treated subjects showing an improvement of at least 2.50 mm (Fig. 3a, b) for both the right and the left side of the arches. “BRs” were defined as those treated subjects showing no improvement (0 mm), an improvement less than 2.50 mm, or worsening of the molar relationship (Fig. 3c, d) for both the right and the left side of the arches. GR consisted of 18 subjects, 10 females and 8 males, whereas BR comprised 12 subjects, 7 females and 5 males.

### Sample size calculation and method error analysis

Sample size of at least 10 subjects per group was necessary to detect a power of 0.8. Sample size was calculated on the measurements of three patients per group selecting as main outcome mandibular length Co-Gn (mm). Thirteen randomly selected cephalograms were retraced by the same operator (CB). No significant mean differences between the two series of records were found by using paired *t* test. Dahlberg’s formula [20] was used to establish the method error. A range from 0.5 to 1.1 mm for linear measurements and 0.6° to 1.3° for angular measurements was found. Reliability coefficient (*r*) ranged from 0.91 to 0.96 respectively. Method error was also calculated for the inclusion in GR group or BR group according to the measurement of molar Class II on the dental casts. Dental casts of 10 randomly selected patients were measured a second time by the same trained operator (CB), and comparison between the first and second registration was performed using Pearson rho correlation coefficient. The two recordings showed a rho value of 0.96.

### Statistical analysis

SPSS software, version 22.0 (SPSS® Inc., Chicago, Illinois, USA), was employed to perform statistical analysis. Parametrical methods were used after having tested the normality of distributions (Shapiro-Wilk test) and equality of variances (Levene’s test) between the groups (GR and BR). Means and standard deviations (SDs) were computed for all tested variables, and Student’s *t* test was used to assess significance of the differences between groups (GR and BR). A *P* value less than 0.05 was used in rejection of the null hypothesis.

Discriminant analysis [21] was applied to cephalometric values of the 30 subjects at T1. All the assumptions were verified before application of discriminant analysis as follows: (i) number of tested variables did not exceed *n −* 2, where *n* is the sample size of the smaller group; (ii) normal distribution and equality of the variances; (iii) non-multicollinearity of the variables; and (iv) absence of outliers, verified with the interquartile range (IQR) method. Eight variables were used as predictors in the discriminant analysis: Ar^Go^N, N^Go^Me, N^S^Ar, Co^Gn, S^Ar, Ar^Go, Go^Me, and S^Ar^Go. The first phase of the analysis was to detect the most important variables for group separation between GR and BR by means of stepwise variable selection. Forward selection procedure with F-to-enter and F-to-remove equal to 4 was chosen. When the smallest set of significant discriminant variables was selected, the predictive power (classification power) of the model was tested with discriminant analysis.

## Results

GR patients showed an improvement of 2.99 ± 0.45 mm (mean ± SD), and BR patients showed changes of 1.02 ± 0.53 mm (mean ± SD) in molar evaluation on the dental casts in average between the right and the left side.

Means, SDs, and *P* values of cephalometric measurements are reported in Table 2. Sagittal measurements showed no significant differences between the two groups. Among the vertical measurements, articular angle (S^Ar^Go) showed significant reduced values in BR group (140.85 ± 4.22°) when compared to GR group (144.66° ± 4.81°). On the contrary, superior gonial angle (Ar^Go^N) showed significant greater values in BR group (55.26° ± 3.48°) than in GR group (53.01° ± 2.46°). All mandibular dimensions (Co-Gn, S-Ar, Ar-Go, Go-Me) showed significant reduced values in GR group when compared to BR group.

**Table 2:** Comparison between BR and GR groups

	BR group		GR group		
	Mean	SD	Mean	SD	P
Age	8.32	1.10	8.29	1.06	0.945
SNA (°)	81.65	2.57	80.60	4.33	0.457
SNB (°)	76.79	2.01	75.01	4.62	0.223
ANB (°)	4.87	2.32	5.38	1.96	0.520
ANPg (°)	4.18	2.43	4.78	2.28	0.497
Wits (mm)	1.97	3.88	1.03	2.76	0.447
OVJ (mm)	6.82	3.53	5.04	1.94	0.091
AnsPns^GoGn (°)	22.83	3.31	24.29	4.67	0.356
SN^GoGn (°)	30.18	3.16	33.23	5.44	0.092
OVB (mm)	3.22	1.94	2.74	1.74	0.496
Ar^Go^N (°)	55.26	3.48	53.01	2.36	0.048*
N^Go^Me (°)	70.28	3.69	71.84	4.78	0.354
N^S^Ar (°)	125.38	3.95	124.94	4.97	0.804
S^Ar^Go (°)	140.85	4.22	144.66	4.81	0.036*
Sum. Jaraback (°)	391.77	3.58	394.46	6.79	0.222
Co-Gn (mm)	103.68	5.83	95.36	6.31	0.001**
S-Ar (mm)	32.59	3.75	28.93	3.18	0.008**
Ar-Go (mm)	41.17	3.99	37.49	2.75	0.006**
Go-Me (mm)	63.36	5.45	57.63	5.05	0.006**

Data are shown as mean and SDs. Student’s *t* test was used to assess significance of the differences between groups, and significance levels are shown in *P* column

*BR* bad responder, *GR* good responder

*P < 0.05, **P < 0.01

**Fig. 4:**
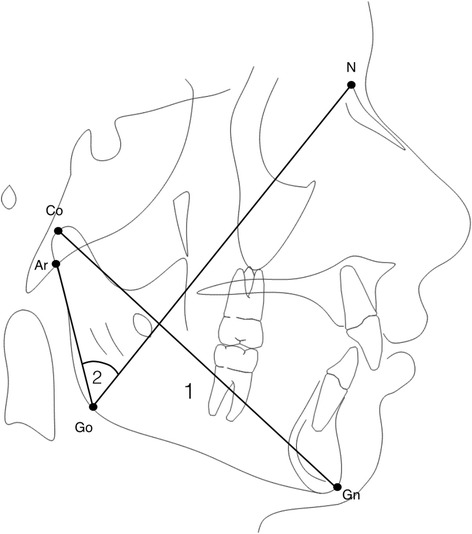
Predictive measurements of successful improvement of Class II after RME treatment. *1* mandibular length (Co-Gn), *2* superior gonial angle (Ar^Go^N)

Stepwise variable selection generated a two-variable model that produced the most efficient separation between the two groups (GR vs BR). The variables selected were the mandibular length (Co-Gn) and the superior gonial angle (Ar^Go^N) (Fig. 4 and Table 3). The classification power of the selected two-variable model was 83.3% (Table 4). Only one out of five cases in each group was not classified correctly. Unstandardized discriminant function coefficients of the selected variable together with a calculated constant (Table 5) lead to the following equation that provides individual scores for the assignment of a new case to GR or to BR:

**Table 3:** Stepwise variable selection procedure

Variables in model	F-to-remove = 4	Variables not in model	F-to-enter = 4
Co-Gn	13.797	N^Go^Me	0.446
Ar^Go^N	5.298	N^S^Ar	0.130
		S^Ar^Go	5.270
		S-Ar	8.278
		Ar-Go	8.970
		Go-Me	8.686
Wilks lambda = 0.567*			

Among the cephalometric variables, only Ar^Go^N, N^Go^Me, N^S^Ar, Co^Gn, S^Ar, Ar^Go, Go^Me, and S^Ar^Go were selected for discriminant analysis. The variables selected from the stepwise variable selection were the mandibular length (Co-Gn) and the superior gonial angle (Ar^Go^N). Significance of Wilks lambda was set at *P* < 0.05

*P < 0.05

**Table 4:** Discriminant analysis

		Predicted group membership			
		GR		BR	
Group	No. of cases	*n*	%	*n*	%
Group GR (success)	12	10	83.3	2	16.7
Group BR (failure)	18	3	16.7	15	83.3

Percentage of cases correctly classified 83.3%. Classification results of discriminant analysis

**Table 5:** Discriminant function

Predictive variables	Unstandardized canonical discriminant function coefficients
Co-Gn	0.147
Ar^Go^N	0.221
Constant	−26.399

Individual Score = 0.147_(Co-Gn)_ + 0.221_(Ar^Go^N)_ − 26.399. Discriminant scores for group means (group centroids): successful group = −0.690; unsuccessful group = 1.034; and critical score = 0.667. Unstandardized discriminant function coefficients of the selected variable together with a calculated constant (−26.399) lead to the equation that provides individual scores for the assignment of a new case to GR or to BR

$$ \mathrm{Individual}\kern0.5em \mathrm{Score}=\kern1em 0.14{7}_{\left(\mathrm{Co}\hbox{-} \mathrm{Gn}\right)}+\kern0.62em 0.22{1}_{\left(\mathrm{A}{\mathrm{r}}^{\wedge}\mathrm{G}{\mathrm{o}}^{\wedge}\mathrm{N}\right)}-26.399 $$

The critical score (i.e., the value dividing GR from BR) is 0.667, i.e., the mean value of the group centroids of the two groups (−0.690 and 1.034 for GR and BR, respectively) (Table 5). Each new patient with dental Class II malocclusion at CS 1–2 who will show an individual score smaller than the critical score is expected to respond favorably to RME treatment in terms of at least 2.50 mm of improvement in molar relationship. On the contrary, each new patient with dental Class II malocclusion at CS 1–2 who will show an individual score greater than the critical score is expected to have a poor response to RME treatment in terms of at least 2.50 mm of improvement in molar relationship.

## Discussion

Several studies investigated possible “spontaneous” correction of Class II malocclusion after maxillary expansion; nevertheless, these studies present different methodology and controversial results [22, 23]. To the best of our knowledge, this is the first study that differentiates bad and good responders in improving occlusal relationship after palatal expansion. Previous studies investigated Class II patients measuring changes after treatment without making any differences between patients who improved and who did not improve malocclusion [7, 8, 13]. This study design might not allow to evidence possible variables influencing improvement, since mean changes might not reach clinical significance; in fact, they are the result of average of patients who improved and who showed no improvement pooled together. Present study design, separating GR from BR, allowed detecting significant differences between the two groups. Indeed, according to the results of present investigation, the amount of dental and skeletal Class II does not seem a discriminant variable in influencing improvement. In fact, patients with similar sagittal measurements of skeletal Class II (ANB, ANPg in Table 2) might show improvement of the malocclusion after RME or not. On the contrary, mandibular lengths and mandibular sagittal position showed significant differences in comparisons between groups. GRs showed statistically significant greater articular angle, more acute superior gonial angle, and reduced mandibular dimensions (Co-Gn, S-Ar, Ar-Go, Go-Me) when compared to BRs.

Discriminant analysis confirmed mandibular dimension (Co-Gn) and superior gonial angle (Ar^Go^N) as cephalometric variables with significant predictive value in assigning patients to one group or the other. In particular, patients with smaller mandibular length and more acute superior gonial angle showed significant improvement of Class II malocclusion after RME. Smaller mandibular length might show additional potential growth, which could take place after correction of transverse maxillary deficiency showing sagittal improvement of Class II malocclusion. In addition, successful patients showed more acute superior gonial angle which might be due to forward position of Ar landmark, suggesting forward position of the glenoid fossa improving the prognosis of Class II malocclusion. Moreover, a distal position of the mandible, confirming the more distal position of glenoid fossa, in BR group was suggested by a significantly lower articular angle (S^Ar^Go), increased superior gonial angle (Ar^Go^N), and increased S-Ar distance when compared to GR group. Unfortunately, scientific evidence suggesting whether dental correction or mandibular anterior shift and/or supplementary growth take place in Class II individuals after RME is still lacking [23]. Some authors reported significant occlusal sagittal improvements after RME [24, 25]. Nevertheless, these investigations could present some limitations related to time interval observation comprising transition from mixed to permanent dentition, which might have influenced occlusal improvements related to position of the first permanent molars. In order to avoid these confounding factors, we included in our study only early mixed dentition patients at both the evaluation time points. Since occlusal changes did not occur in the present sample, because patients without discrepancy between CR and CO nor with changes in dentition during the time interval were selected, Class II improvement after RME could be related to mandibular growth or anterior shift. Unfortunately, cephalograms at T2 were not available for the tested sample due to ethical reasons, and this might be considered as a limit of the present study since the Class II improvement was measured on dental casts only. Nevertheless, the comprehension of reasons for improvement was not the aim of the present study.

The rigidity of applied inclusion and exclusion criteria led to a small final sample compared to the initial sample of the present study. This rigid selection might have caused selection bias, which might be consider as a limit of the present investigation, but on the other hand, this methodology assured a great homogeneity among the selected patients which is challenging in a retrospective study. This homogeneity in patients’ selection was considered of great importance since RME treatment might have caused high variation in individual response.

Some authors have reported that maxillary expansion might be detrimental for correction of Class II malocclusion, due to downward and backward displacement of mandible that frequently occurs after RME [10–12] caused by extrusion due to buccal tipping of the first upper molars involved in expansion appliance [26]. Different treatment outcomes might be related to collateral function such as changes in the breathing pattern [27–29], different mandibular displacement [30, 31], and/or spontaneous dental changes in the lower arch [32], but these variables were not evaluated in the present study. The present study employed maxillary expander banded on the upper second primary molars [33, 34], and results should be limited to this appliance. Unfortunately, none of the previous cited studies evaluated mandibular response in Class II patients after RME on the upper second primary molars, but Rosa et al. [31] suggested spontaneous changes in mandibular position in Class III patients with the use of this appliance. Since the upper permanent molars are not anchored in the appliance and are free to move within the occlusal forces, spontaneous movement and distal rotation [26] might have occurred allowing forward placement of mandible after treatment.

Considering clinical importance of outcomes although the limitation of present retrospective design, further studies conducted with prospective design are necessary to confirm present results.

## Conclusions

- The assessment of spontaneous improvement of Class II malocclusion after RME therapy was performed by means of discriminant analysis, to identify a significant model of predictive variables. Two predictive measurements were selected: (1) length of mandible (Co-Gn) and (2) superior gonial angle (Ar^Go^N).
- The classification power of the model for predicting success or failure is 83.3% for each new patient. Spontaneous correction of Class II malocclusion after RME in early mixed dentition might be favorable when patient’s cephalometric records show decreased mandibular length and more acute superior gonial angle at the start of treatment.
- The important role of mandibular dimensions and mandibular sagittal position in diagnostic and prognostic evaluation of Class II patients deserves to be emphasized, suggesting poor response when increased mandibular dimensions and more distal position of the mandible are identified in pretreatment cephalograms. Class II skeletal angular measurements before treatment are not able to improve this prediction based upon mandibular dimensions and superior gonial angle.

## Abbreviations

BR: Bad responder
CO: Centric occlusion
CR: Centric relation
CVM: Cervical vertebral maturation
GR: Good responder
NHP: Natural head position
RME: Rapid maxillary expansion
SD: Standard deviation
